# Interlobar Pulmonary Hamartoma With an Unusual Neighboring Lesion: A Case Report

**DOI:** 10.7759/cureus.14008

**Published:** 2021-03-20

**Authors:** Mohamed Abdelghaffar, Fatima Mandeel, Zahraa Rabea, Sara George, Mahmood Al Saeed

**Affiliations:** 1 Pulmonary Medicine, Salmaniya Medical Complex, Manama, BHR; 2 Internal Medicine, Salmaniya Medical Complex, Manama, BHR; 3 Pathology and Laboratory Medicine, Salmaniya Medical Complex, Manama, BHR

**Keywords:** benign lung tumors, hamartoma, pulmonary hamartoma, sclerosing pneumocytoma

## Abstract

Pulmonary hamartomas are benign lung tumors. They are uncommon and represent a small percentage of all solitary lung lesions. Hamartomas are composed of an abnormal mixture of epithelial and mesenchymal elements. They are usually discovered incidentally as patients are asymptomatic in most cases. On the other hand, sclerosing pneumocytomas are rarely discovered and are one of the most uncommon benign lung tumors. Herein, we present the case of a 37-year-old female who presented with hemoptysis. A computed tomography scan of her thorax revealed a right-sided round lesion in the interlobar fissure with no additional findings. Surgical excision was performed, which demonstrated an uncommon and unique finding of a pulmonary hamartoma coexisting with a sclerosing pneumocytoma found on exploration. This highlights the importance of exploration during surgical procedures in order identify missed lesions on imaging.

## Introduction

Pulmonary hamartoma (PH), also known as mesenchymoma, was first described by Albrecht in 1904 [1]. In 1934, Goldsworthy applied this term to benign tumors located in the lung that were predominantly a combination of fat and cartilage [2]. PHs usually present in the fifth and sixth decades of life with a 4:1 male to female ratio [3]. They are usually detected incidentally on chest radiography, presenting with a coin lesion appearance. PHs are uncommon and make up 8% of all solitary lung lesions; however, they are considered the commonest benign pulmonary nodules (77%) [4]. They most frequently occur within the lung parenchyma. Pulmonary sclerosing pneumocytoma (PSP) is a rare benign lung tumor. A study by Chung et al. showed that PSPs mostly affect women in the fifth decade and most patients are asymptomatic with lesions discovered incidentally on chest radiography as a solitary nodule or mass [5]. We report a unique case of a 37-year-old female who presented with a history of hemoptysis. Routine chest x-rays revealed a round shadow in a loculated interlobar effusion. Computed tomography (CT) of the thorax was performed which confirmed the presence of an encysted effusion along the superior aspect of the superior oblique fissure containing a solitary lesion within. There were no additional findings. The lesion was surgically excised and another neighboring mass was also identified at the time of surgery. Histological examination revealed a PH coexisting with a PSP of angiomatous type. Our case is, therefore, rare in terms of its location, age of presentation, and coexistence of another unusual tumor. It also highlights the need for performing a thorough exploration during surgical procedures in order to identify additional missed lesions on imaging [6].

## Case presentation

**Figure 1:**
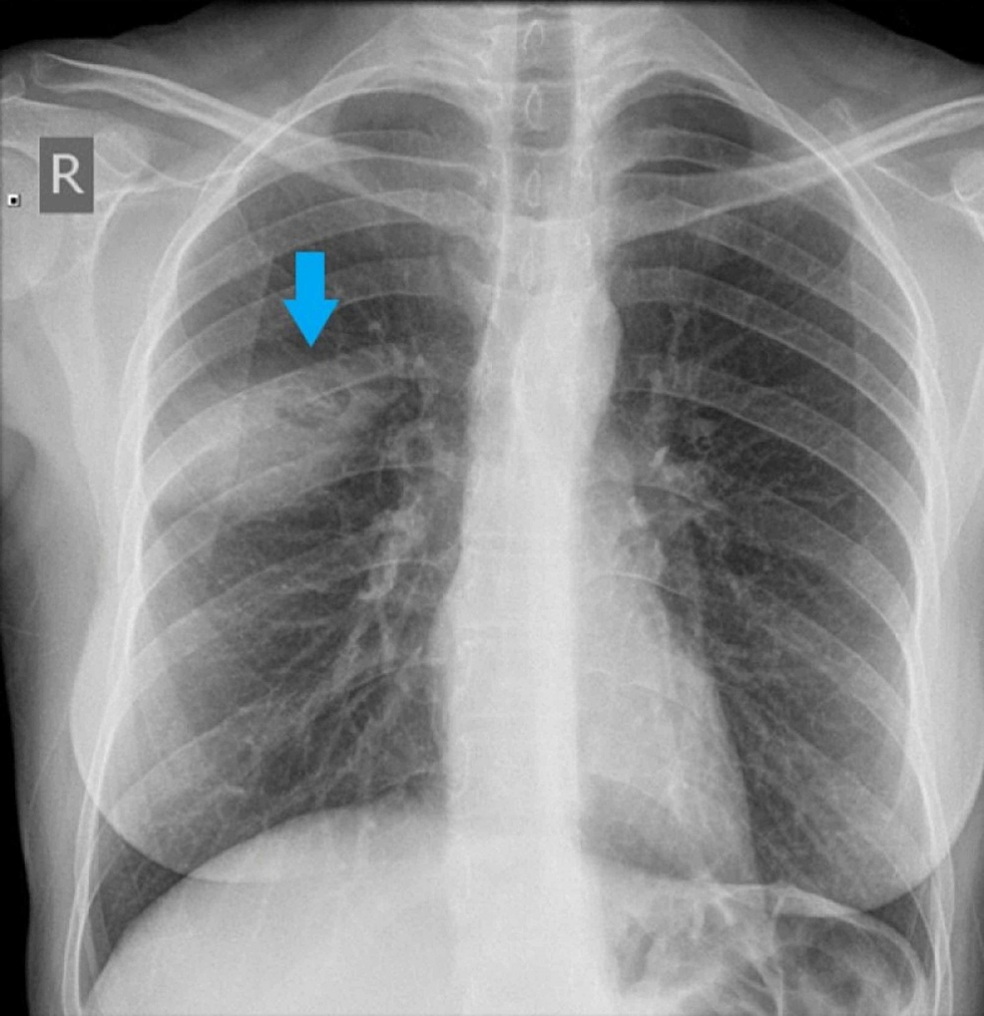
Chest radiograph on admission showing a round opacity in the interlobar fissure of the right lung (arrow)

Our patient is a 37-year-old female who presented with a three-day history of dry cough associated with hemoptysis. The hemoptysis started a day before presentation to the hospital and it occurred twice. The patient quantified the amount equal to a teaspoon full on each instance. She was otherwise asymptomatic with no associated fever, night sweats, or weight loss. Physical examination was completely unremarkable with clear lung fields on auscultation. Laboratory investigations, including a full blood count, renal function, electrolytes, and inflammatory markers, were also within normal limits. Her past medical history was unremarkable and she never smoked cigarettes. There was no additional history of pets at home or occupational exposures. Plain chest radiography revealed a right middle zone elliptical opacity with a small round shadow within (Figure 1). She was admitted for further investigations and treated symptomatically.

**Figure 2:**
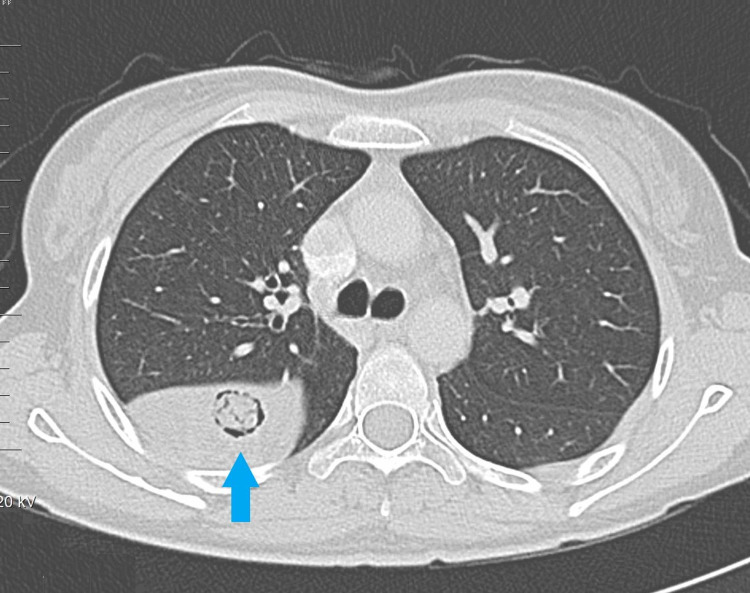
Computed tomography scan shows a loculated effusion in the right oblique fissure embedded inside it a well-defined, rounded, soft tissue density lesion measuring approximately 7 x 5 cm with no calcification

A CT scan was performed which demonstrated a loculated effusion in the upper part of the oblique fissure with a well-defined, rounded, soft tissue density lesion embedded within and measuring approximately 7 x 5 cm without calcifications (Figure 2). 

This case was later discussed at a multidisciplinary team meeting followed by a plan to proceed with exploration and excision of the lesion.

**Figure 3:**
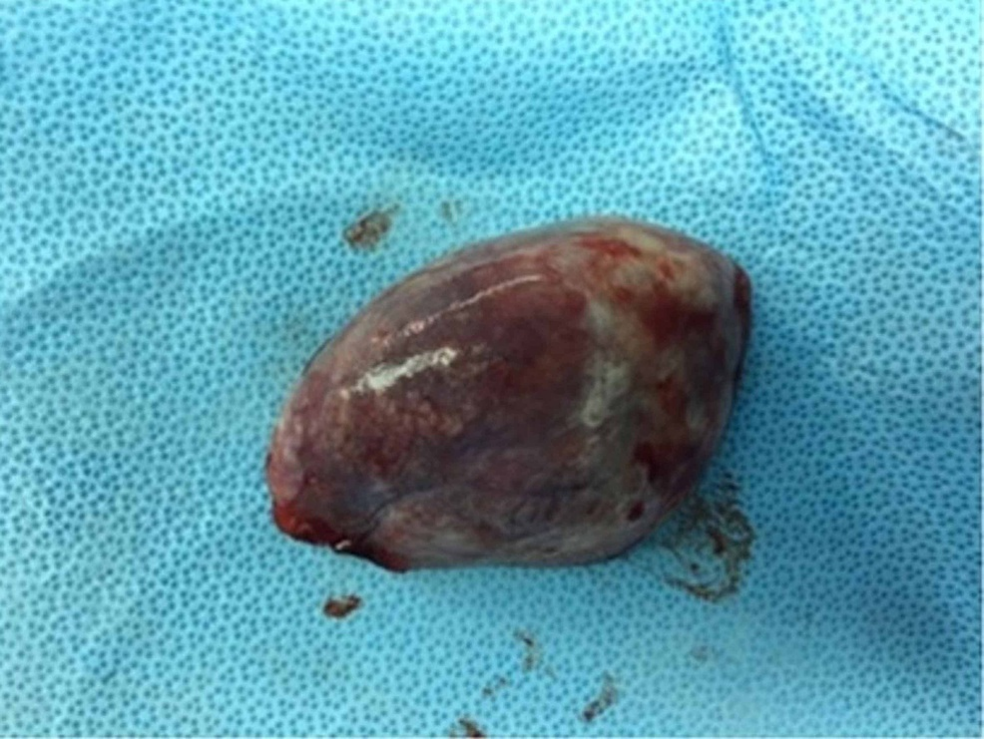
The gross appearance of the excided primary lesion seen on computed tomography and x-ray

Surgery was initially performed by the means of a thoracoscopy through a 12 mm port. The interlobar fissure cyst containing the lesion was large in size (10 x 7 cm); therefore, it proved to be difficult to remove while keeping the cyst intact by thoracoscopy. This was important in order to avoid a sac rupture with the remote possibility of it being a hydatid cyst. Eventually, a right posterolateral thoracotomy through the fifth intercostal space was performed and the cyst dissected with complete excision (Figure 3).

Surprisingly, during exploration, another mass was noted in the upper lobe ipsilaterally and was excised. The wound was then closed and both lesions were sent to the laboratory for histopathological examination. There were no immediate complications and the recovery period postoperatively was unremarkable, alongside a complete resolution of the hemoptysis and cough.

**Figure 4:**
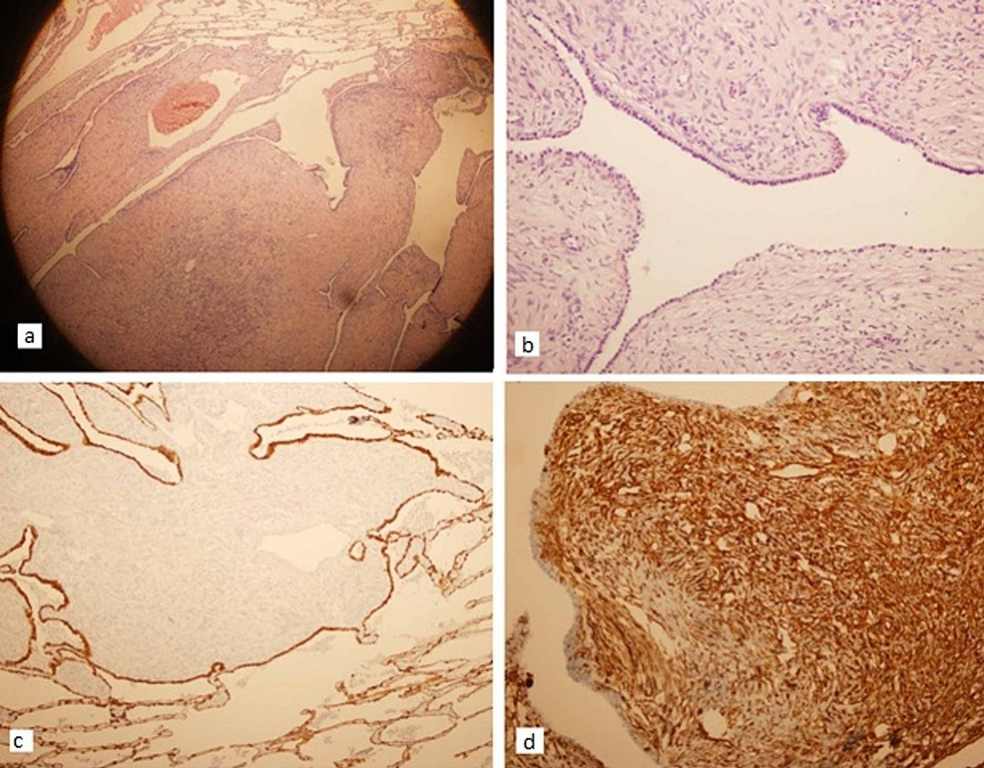
Histopathology of the primary lesion consistent with pulmonary hamartoma a) scanner view shows the pulmonary hamartoma with adjacent lung tissue at the periphery. The lesion shows broad papillary excrescences covered by simple epithelium (hematoxylin & eosin (H&E) x40); b) broad papillary excrescences covered by cuboidal epithelium and stroma showing spindle cells (H&E x200); c) surface epithelium of the pulmonary hamartoma, staining positive for cytokeratin (immunohistochemical stain CK7 with haematoxylin counterstain x100); d) stromal cells of the pulmonary hamartoma positive for vimentin (immunohistochemical stain for vimentin with haematoxylin counterstain x200).

The first soft tissue lesion resected from the interlobar fissure measured 2 x 1.5 x 1 cm. Its histological findings were consistent with a pulmonary hamartoma (Figure 4). 

**Figure 5:**
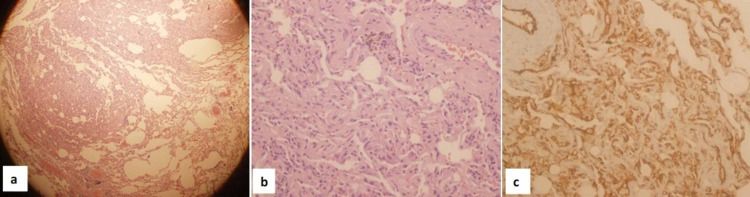
Histopathology of the second lesion consistent with sclerosing pneumocytoma - angiomatous type a) scanner view showing an ill-defined area of thin-walled blood vessels constituting the sclerosing pneumocytoma-angiomatous type (hematoxylin & eosin (H&E) x40); b) multiple thin-walled blood vessels with mild smooth muscle proliferation (H&E stain, x400); c) endothelium of the blood vessels positive for CD31 (immunohistochemical stain for CD31 with haematoxylin counterstain x400).

The second lesion that was found on exploration in the upper lobe ipsilaterally measured 1 x 1 x 0.3 cm. On microscopic examination, it showed an ill-defined area of multiple thin-walled blood vessels with mild focal smooth muscle proliferation. The vessels showed positive immunohistochemical staining for the vascular endothelial marker. This was consistent with the diagnosis of sclerosing pneumocytoma, the angiomatous type (Figure 5).

## Discussion

Pulmonary hamartomas consist of a disorganized array of various connective and epithelial tissue normally found in the lungs. In a study by Cosio et al., it was concluded that they are the most common form of benign lung tumors, are male predominant, and peak in incidence in the fifth or sixth decade of life [7]. They are usually asymptomatic and only occasionally cause symptoms, such as bronchial obstruction leading to atelectasis, cough, sputum production, and chest pain [4]. Our patient was female, in her late thirties, and had symptoms of hemoptysis, cough, and upper respiratory tract infection. Over 90% of the tumors are peripheral, and 10% or less are endobronchial; moreover, peripheral tumors constitute 7% - 14% of all radiographic solitary pulmonary nodules [8]. On CT scan, these lesions are described as a rounded soft tissue mass that frequently exhibits calcifications and densities of adipose tissue [4]. Our case demonstrates a PH originating from the interlobar fissure which is extremely rare and, to the best of our knowledge, is not documented in the literature.

The mesenchymal components of PH are highly variable in histology and are composed of a mixture of mature mesenchymal tissue [4]. They are usually slow-growing and malignancy should always be ruled out when encountering such a lesion [4]. In a study by Gjevre et al., it was concluded that surgical resection is usually curative with a very low chance of recurrence [9].

PSP is a rare finding accounting for only 1% of all benign lung tumors with a female predominance and a peak incidence in the fifth decade [10]. Most patients are asymptomatic; however, some present with dyspnea, cough, hemoptysis, and chest pain [10-12]. It is usually discovered incidentally on imaging as a solitary, well-circumscribed, juxta-pleural nodule or mass that, on CT examination, is characterized by intense and homogenous enhancement due to its hemangiomatous component [5].

Both of these tumor types are composed of two kinds of cells. These include cuboidal epithelium lining cells and round cells with a variable architecture, including papillary, sclerotic, solid, and hemorrhagic. Molecular studies indicate that PSPs are true tumors, unlike a typical hamartoma [12]. PSP can manifest as multiple tumors in up to 4% of cases [13]. Surgical resection allows for histological diagnosis with characteristic immunostaining patterns in the majority of cases [14]. The prognosis after surgical resection is excellent, and although it is generally a benign lesion, follow-up is necessary due to the low chance of recurrence [11].

## Conclusions

Herein, we report an unusual case of PH. Our patient is a young female as opposed to the common presentation of males in their fifties or sixties. The location is also unusual with an extremely rare finding of a neighboring PSP. PHs are benign, rare, and usually present as a solitary pulmonary nodule on chest radiography. Definite diagnosis and treatment can be achieved by surgical resection as it is important to exclude a possible malignancy. In addition to that, proper visualization and exploration of the surrounding area in relation to the primary lesion may help discover a second hidden lesion, as demonstrated in our case.
